# Volumetric‐modulated arc therapy for the treatment of a large planning target volume in thoracic esophageal cancer

**DOI:** 10.1120/jacmp.v14i3.4269

**Published:** 2013-05-06

**Authors:** Ahmar S. Abbas, Douglas Moseley, Zahra Kassam, Sun Mo Kim, Charles Cho

**Affiliations:** ^1^ Stronach Regional Cancer Centre Newmarket ON Canada; ^2^ Princess Margaret Cancer Centre Toronto ON Canada; ^3^ Department of Radiation Oncology University of Toronto ON Canada

**Keywords:** IMRT, VMAT, monitor units, delivery time, integral dose

## Abstract

Recently, volumetric‐modulated arc therapy (VMAT) has demonstrated the ability to deliver radiation dose precisely and accurately with a shorter delivery time compared to conventional intensity‐modulated fixed‐field treatment (IMRT). We applied the hypothesis of VMAT technique for the treatment of thoracic esophageal carcinoma to determine superior or equivalent conformal dose coverage for a large thoracic esophageal planning target volume (PTV) with superior or equivalent sparing of organs‐at‐risk (OARs) doses, and reduce delivery time and monitor units (MUs), in comparison with conventional fixed‐field IMRT plans. We also analyzed and compared some other important metrics of treatment planning and treatment delivery for both IMRT and VMAT techniques. These metrics include: 1) the integral dose and the volume receiving intermediate dose levels between IMRT and ${\text{VMAT}}_{I}$ plans; 2) the use of 4D CT to determine the internal motion margin; and 3) evaluating the dosimetry of every plan through patient‐specific QA. These factors may impact the overall treatment plan quality and outcomes from the individual planning technique used. In this study, we also examined the significance of using two arcs vs. a single‐arc VMAT technique for PTV coverage, OARs doses, monitor units and delivery time. Thirteen patients, stage T2‐T3 N0‐N1 (TNM AJCC 7th edn.), PTV volume median 395 cc (range 281–601 cc), median age 69 years (range 53 to 85), were treated from July 2010 to June 2011 with a four‐field (n=4) or five‐field (n=9) step‐and‐shoot IMRT technique using a 6 MV beam to a prescribed dose of 50 Gy in 20 to 25 F. These patients were retrospectively replanned using single arc (${VMAT}_{I}$, 91 control points) and two arcs (${VMAT}_{II}$, 182 control points). All treatment plans of the 13 study cases were evaluated using various dose‐volume metrics. These included PTV D99, PTV D95, PTV $V95_{47.5Gy}(95\%)$, PTV mean dose, $D_{\text{max}}$, PTV dose conformity (Van't Riet conformation number (CN)), mean lung dose, lung V20 and V5, liver V30, and $D_{\text{max}}$ to the spinal canal prv3mm. Also examined were the total plan monitor units (MUs) and the beam delivery time. Equivalent target coverage was observed with both VMAT single and two‐arc plans. The comparison of ${\text{VMAT}}_{I}$ with fixed‐field IMRT demonstrated equivalent target coverage; statistically no significant difference were found in PTV D99 (p=0.47), PTV mean (p=0.12), PTV D95 and PTV $V95_{47.5Gy\;(95\%)}\;(p=0.38)$. However, $D_{\text{max}}$ in ${\text{VMAT}}_{I}$ plans was significantly lower compared to IMRT (p=0.02). The Van't Riet dose conformation number (CN) was also statistically in favor of ${\text{VMAT}}_{I}$ plans (p=0.04). ${\text{VMAT}}_{I}$ achieved lower lung V20 (p=0.05), whereas lung V5 (p=0.35) and mean lung dose (p=0.62) were not significantly different. The other OARs, including spinal canal, liver, heart, and kidneys showed no statistically significant differences between the two techniques. Treatment time delivery for ${\text{VMAT}}_{I}$ plans was reduced by up to 55% (p=5.8E−10) and MUs reduced by up to 16% (p=0.001). Integral dose was not statistically different between the two planning techniques (p=0.99). There were no statistically significant differences found in dose distribution of the two VMAT techniques (${VMAT}_{I}$ vs. ${VMAT}_{II}$) Dose statistics for both VMAT techniques were: PTV D99 (p=0.76), PTV D95 (p=0.95), mean PTV dose (p=0.78), conformation number (CN) (p=0.26), and MUs (p=0.1). However, the treatment delivery time for ${\text{VMAT}}_{\text{II}}$ increased significantly by two‐fold (p=3.0E−11) compared to ${VMAT}_{I}$. VMAT‐based treatment planning is safe and deliverable for patients with thoracic esophageal cancer with similar planning goals, when compared to standard IMRT. The key benefit for ${\text{VMAT}}_{I}$ was the reduction in treatment delivery time and MUs, and improvement in dose conformality. In our study, we found no significant difference in ${\text{VMAT}}_{\text{II}}$ over single‐arc ${\text{VMAT}}_{I}$ for PTV coverage or OARs doses. However, we observed significant increase in delivery time for ${\text{VMAT}}_{\text{II}}$ compared to ${VMAT}_{I}$.

PACS number: 87.53.Kn, 87.55.‐x

## I. INTRODUCTION

Recent technological advances in radiation treatment (e.g., intensity‐modulated radiotherapy, image‐guided radiotherapy) have progressively changed the practice in esophageal cancer.^1^, ^2^ These new technological developments in radiation therapy help to improve target dose coverage while reducing the organs‐at‐risk doses, and improve the precision in delivering accurate radiation doses to the tumor while minimizing the risk of damaging surrounding healthy tissues.

In this retrospective planning and delivery study, we evaluate the feasibility of volumetric‐modulated arc therapy (VMAT) as a technique to address some of the limitations of fixed‐field intensity‐modulated radiation therapy (IMRT) treatments for the large planning target volumes (PTVs) of distal esophageal cancers. In a standard IMRT treatment planning technique, the selection of beam angles can be challenging for large PTV volumes, and it is often difficult to achieve the desired PTV dose coverage by limiting the organs‐at‐risk doses. The major conceptual advantage of modulated arc therapy over standard fixed‐field IMRT techniques is that the radiation source (gantry) is rotating around the patient when delivering radiation. Thus all angles are available to deliver radiation to the target while avoiding critical structures, and the delivery time is used efficiently because the radiation delivery does not stop in between different beam angles.^3^, ^4^ Whereas for standard step‐and‐shoot IMRT, delivery of all static beams would take a longer time to complete the treatment.

## II. MATERIALS AND METHODS

Radiation plans of 13 patients with distal esophageal cancer, stage T2‐T3 N0‐N1 (TNM AJCC 7th edn.), treated between July 2010 and June 2011, with four‐field (n=4) or five‐field (n=9) 6 MV IMRT techniques, to a prescribed dose of 50 Gy in 20 to 25 fractions, were retrospectively reviewed. In all clinical IMRT plans, selection for the number of the treatment beams, gantry angles, and the optimization parameters were based on the clinical objectives to achieve the desired PTV coverage and better sparing of OARs.

This study received institutional ethics approval. Our standard institutional protocol requires all patients with distal esophageal cancer to be scanned with a helical voluntary exhale breath‐hold CT scan for volume delineation and dose computation, followed by a 4D CT for ITV generation. All patients were scanned on Philips Brilliance Big Bore CT (Philips Healthcare, Andover, MA). This device provides up to a 60 cm field of view. The 4D CT scans were captured using a shallow helical acquisition and a respiratory sensor (air bellows belt). Prior to the CT scan, the respiratory sensor is placed on the patient's chest or abdomen. The sensor measures the respiration trace of the patient during acquisition. This respiration signal is then phase‐binned into tem bins. The captured sinograms are then reconstructed into each of the phase bins using the CT scanners’ reconstruction system. The maximum inhale and maximum exhale datasets are then selected and sent to the treatment planning system for delineating the CTVs and ITV. No oral contrasts were used, unless requested by a radiation oncologist. Standard planning target volumes included GTV, CTV, ITV, and PTV. The contoured critical organs included lungs, kidneys, heart, spinal canal, and liver. GTV and CTV were contoured on both primary helical CT dataset and 4D CT maximum exhale and maximum inhale sequences. The CTV volumes were adjusted to respect the natural anatomic barriers such as bone and the lung/air interfaces, where there would be no expectation of microscopic extension of disease. The CTV on 4D CT sequences and on primary helical CT scans were combined to form an ITV. The PTV was created by using a 0.5 cm isotropic expansion from the ITV. All other volumes at risk were contoured on the primary helical voluntary exhale breath‐hold scan.

In this study, we also examined the significance of using two full‐arc ${\text{VMAT}}_{\text{II}}$ plans (two coplanar beams each 360° arc, 182 control points) vs. single‐arc ${\text{VMAT}}_{I}$ (single coplanar 360° arc, 91 control points). Similar dosimetric metrics of PTV and OARs were adopted for both ${\text{VMAT}}_{I}$ and ${\text{VMAT}}_{\text{II}}$ as were used with the clinical IMRT plan acceptance criteria.

All IMRT and VMAT plans were generated on Pinnacle^3^ version 9.0revB (Philips Oncology Systems, Madison, WI) treatment planning system. Heterogeneity corrections are applied for all treatment plans dose calculations. PTV coverage of 95% was required, with acceptable hot spots of +5%. Plan comparison was based on PTV coverage and organs‐at‐risk doses. Dose constraints used for organs at risk were: liver V30 — 60% to receive <30 Gy and mean liver dose <30 Gy; heart V45 — 60% to receive <45 Gy; both lungs V20 — 30% to receive <20 Gy, lung V5 — 60% to receive <5 Gy, and mean lung dose <18 Gy; each kidney V22.5 — 33% to receive <22.5 Gy and spinal canal 3 mm‐prv maximum point dose <45 Gy.

Similarly, the objectives and optimization parameters in VMAT single‐ or two‐arc planning techniques were set similar to that of IMRT in order to minimize any systematic biases in the two planning techniques. In addition, in both ${\text{VMAT}}_{I}$ and ${\text{VMAT}}_{\text{II}}$ plans, the substantial numbers of iterations were set to test the optimum solution provided by the optimizer.

All VMAT treatment plans were reviewed by two radiation oncologists (CC and ZK) for assessment of the usual dose volume parameters and acceptability of target coverage and doses to OARs.

Conformation number (CN) was calculated with a Van't Riet model:^(5)^

(1) $$\text{CN}‐\left(\frac{TV_{RI}}{TV}\right)\left(\frac{TV_{RI}}{V_{RI}}\right)$$

Here, ${TV}_{RI}$ is the target volume covered by the reference isodose (95%), *TV* is the target volume (PTV), and $V_{RI}$ is the volume of the reference isodose. The CN ranges from 0 to1, where 1 is the ideal value.

### A.1 Integral dose

In our study we reported the integral dose as the sum of all dose voxels times their mass. The integral dose with variable densities requires a complex calculation. For simplicity, we considered a uniform density for the whole body volume. This assumption was made for both the IMRT and VMAT calculation, so it gives a fair relative comparison. A simplified equation of our integral dose is represented as:

(2) $$IntegralDose(J)=\int \int_{V}\int D(x,y,z)\cdot \rho \cdot dV$$

Here, *V* is the body volume, p is the density, and *D* is the absorbed dose.

### A.2 Volume receiving ≥2Gy and ≥5Gy

We also examined the differences in volume (cc) receiving doses of 2 Gy or more between the two planning techniques, since some models of radiation carcinogenesis suggest that dose response is linear until the dose threshold of 6 Gy, where it then reaches a plateau.^6^, ^7^, ^8^

**Figure 1:**
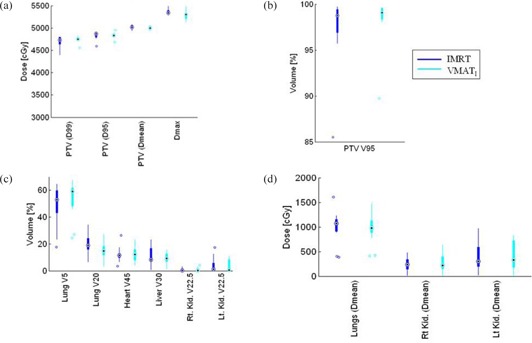
Box‐and‐whisker plot of DVH metrics representation showing the median, max, min, and outliers of IMRT and ${VMAT}_{I}$: (a) PTV (D99, D95, D mean, and $D_{\text{max}}$) dose comparison between IMRT and ${\text{VMAT}}_{I}$ plans; (b) PTV $V95_{47.5Gy(95\%)}$ coverage for IMRT and ${\text{VMAT}}_{I}$ plans; (c) organ at risk volume of lungs, heart, liver, kidneys, and spinal canal 3 mm prv comparison; (d) organ at risk mean doses (lungs, right and left kidney).

Standard box‐and‐whisker plots (Fig. 1) and Wilcoxonrank sum hypothesis (Table 1) were used to compare IMRT and VMAT DVHs samples to determine statistically significant differences in PTV coverage and organs‐at‐risk doses. The integral doses in joules (J) and volume receiving ≥2 Gy and ≥5 Gy, were plotted using a box plot and linear correlation plot (2, 3).

**Table 1:** Statistical comparison of dosimetric parameters for (n=13) fixed‐field IMRT vs. ${\text{VMAT}}_{I}$ (single arc) plans, and ${\text{VMAT}}_{I}$ vs. ${\text{VMAT}}_{\text{II}}$ (two arc) plans.

		*IMRT*				${VMAT}_{I}$				*IMRT vs.* ${VMAT}_{I}$ *p*	${VMAT}_{I}$ *vs.* ${VMAT}_{II}$ *p*
*Target OARs*	*Dose Metrics*	*Median*	*Max.*	*Min.*	*SD*	*Median*	*Max.*	*Min.*	*SD*		
PTV	D99 (Gy)	47.3	48.1	44.1	1.1	47.6	48.4	45.6	0.7	0.47	0.76
	Mean Dose (Gy)	50.2	50.9	49.4	0.4	50.0	50.8	49.4	0.4	0.12	0.78
	D95 (Gy)	48.2	49.3	46.0	0.9	48.3	49.1	47.7	0.3	0.76	0.95
	${V95}_{47.5Gy(95\%)}(\%)$	98.5	99.6	95.0	1.5	99.1	99.6	97.4	0.8	0.38	0.80
	$D_{\max}\;(Gy)$	53.5	54.9	52.9	0.7	52.7	53.8	51.5	0.8	0.02	0.78
	Conf.Number (frac.)	0.8	0.9	0.6	0.1	0.8	0.9	0.8	0.1	0.04	0.26
Both Lungs	Mean (Gy)	10.8	16.2	3.8	3.3	9.8	14.8	4.2	2.9	0.62	0.86
	V20 (%)	19.0	34.4	5.8	7.7	14.5	27.8	3.0	6.4	0.05	0.77
	V5 (%)	53.3	65.3	16.6	15.0	60.4	68.2	24.0	13.9	0.35	0.93
Liver	V30 (%)	9.4	23.6	0.0	7.3	9.6	16.1	0.0	4.8	0.70	0.90
Heart	V45 (%)	11.4	25.8	3.3	5.5	12.4	23.0	3.8	5.9	0.80	0.78
RT Kidney	Mean (Gy)	2.9	11.8	0.2	2.8	2.2	9.6	0.2	2.7	0.96	0.94
	V22.5 (%)	0.0	1.4	0.2	0.6	0.0	4.2	0.0	1.2	0.41	0.88
LT Kidney	Mean (Gy)	3.1	11.6	0.2	3.7	3.8	9.1	0.2	3.3	0.84	0.98
	V22.5 (%)	0.0	1.4	0.2	0.6	0.0	4.2	0.0	1.2	0.64	0.99
3mm prv Canal	Max (Gy)	41.8	45.1	31.7	3.9	42.1	47.8	31.3	4.7	0.47	0.57
Monitor Units	MUs	364.0	448.0	309.0	39.2	313.0	381.0	271.0	25.8	0.001	0.10

**Figure 2:**
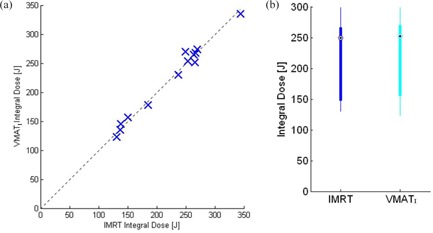
Integral‐dose comparison [J] of ${\text{VMAT}}_{I}$ and IMRT plans: (a) linear plot representing a typical patient dose score; (b) box plot represent the minimal and maximum ranges of integral dose.

**Figure 3:**
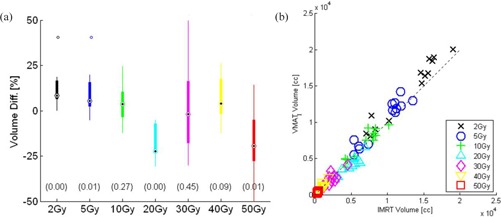
Irradiated volume difference: (a) box plot of ${\text{VMAT}}_{I}$ and IMRT representing percentage of volume differences $\left({VMAT}_{I}-IMRT\right)$ receiving the doses in (Gy); (b) linear plot representing the doses in (Gy) received by the volume in (cc) in ${\text{VMAT}}_{I}$ and IMRT plans.

### B. Delivery time and monitor units

All 13 study cases were tested for beam delivery time. An in‐house MATLAB program (MATLAB, v7.8.0, The MathWorks, Natick, MA) and Dicom vx Listener program were used to record the precise time and monitor the treatment delivery of each beam.

### C. Dosimetry validation

Dosimetry validation of all the 13 study cases for ${\text{VMAT}}_{1}$ and IMRT were tested. All plans were delivered to a 3D diode array phantom (Delta4, ScandiDos, Uppsala, Sweden) using an Elekta Infinity MLCi2 linac (Elekta, Stockholm, Sweden). All IMRT beams were delivered with true gantry angles. The Delta4 phantom consists of 1069 p‐type Silicon diodes in a crossed array inside a cylindrical polymethylmethacrylate (PMMA) phantom. The associated computer software allows comparison of the measured dose distribution for a complete treatment plan with the dose distribution predicted by the treatment planning system. An artificial dataset consisting of a uniform PMMA‐equivalent cylinder is used rather than a real CT scan, so as to avoid errors due to the appearance of the diodes on the CT scan. A relative output calibration for the linac was performed using a 6 MV beam four‐field box. This daily correction factor was generated before the patient plan measurements.

The standard AAPM TG‐119^(6)^ criteria of gamma 3%/3 mm were adopted to review the dosimetric comparison amongst delivered IMRT and ${\text{VMAT}}_{1}$ plans on the Delta4 phantom. Also, an in‐house stringent criteria of dose deviation ±2%, DTA (distance to agreement) ≤2 mm and gamma index ≤1 (max dose dev ±2% and max spatial dev ±2 mm) of 95% pass rate criteria were used for further investigation of significant differences in plan versus dosimetry delivery of both techniques. All ${\text{VMAT}}_{I}$ and IMRT plans were delivered through the MOSAIQ version (2.00U6) record and verify (R&V) system (IMPAC Medical Systems Inc. Sunnyvale, CA) with the most recent release of the console software RT Desktop (V7.0.1) of Elekta Infinity.

## III. RESULTS

**Figure 4:**
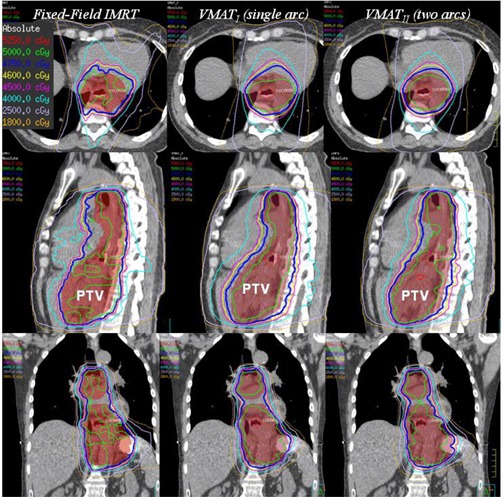
Dose distribution comparison between the IMRT (left), ${\text{VMAT}}_{I}$ single arc (middle), and ${\text{VMAT}}_{\text{II}}$ two full arcs (right) plans for a selected patient, shown in the axial, sagittal, and coronal plane.

The median PTV volume from the 13 patients studied was 395 cc (range 281 to 601 cc). There were five females and eight males, median age 69 years (ranging from 53 to 85) in the study. A typical dose distribution of one of the 13 selected patients for fixed‐field IMRT and VMAT plans is illustrated in (Fig. 4.). Comparison of dosimetric and delivery parameters are summarized in Table 1. All IMRT and VMAT plans met treatment‐planning criteria for plan acceptability. Although there were some differences in dosimetric parameters, it is likely that the IMRT and VMAT treatment plans would be clinically indistinguishable.

### A. ${\text{VMAT}}_{I}$ (single arc) vs. IMRT

The differences between ${\text{VMAT}}_{I}$ and IMRT in dosimetric parameters such as PTV D99 (p=0.47), PTV mean (p=0.12), PTV D95, and PTV ${V95}_{47.5Gy\;(95\%)}\;(p=0.38)$ were not statistically significant (Figs. 1(a) and 1(b)). However, we observed that ${\text{VMAT}}_{I}$ plans had a lower global point $d_{\text{max}}$ (max dose) than IMRT (p=0.02), and visually improved dose homogeneity inside the PTV (Fig. 4) with superior dose conformality (Van't Riet conformation number p=0.04) in favor of ${\text{VMAT}}_{I}$ compared to the IMRT plans. Both lungs’ V20 was also observed in favor of ${\text{VMAT}}_{I}$ plans, p=0.05 (${VMAT}_{I}$ median: 14%, range 3 to 27.8%, and for IMRT plans median: 19%: range 5.8 to 34.4%). However, lung V5 (p=0.35) and mean lung dose (p=0.62) were not significantly different (Figs. 1(c) and 1(d)). Dose to the other OARs met the protocol criteria, with no significant differences between the two planning techniques.

#### A.1 Integral dose

Analysis of the integral dose comparison in joules (J) for all 13 cases are reported in Fig. 2. The linear plot (Fig. 2(a)) represents the dose scores in joules (J) of each ${\text{VMAT}}_{I}$ and IMRT plan, whereas the box plot (Fig. 2(b)) represents the range of integral doses. Statistically, we found no significant difference in integral doses between the two planning techniques (p=0.99).

#### A.2 Volume receiving ≥2Gy and ≥5Gy

We demonstrate the differences in volume (cc) receiving doses of 2 Gy or more between the two planning techniques. Figure 3(a) represents the volume (cc) differences $\left({VMAT}_{I}-IMRT\right)$ receiving several dose metrics, 2 Gy to 50 Gy. Figure 3(b) represents the correlation plot of volume (cc) received by the same doses of 2 Gy or more in ${\text{VMAT}}_{I}$ and IMRT planning techniques. The results of Fig. 3 indicate that the volume (cc) receiving doses between 2 Gy and 5 Gy are less in IMRT (for *2 Gy*, p=0.0002 and for 5 Gy, p=0.01) compared to ${\text{VMAT}}_{I}$ plans and these differences are statistically significant, whereas the volume receiving doses of 20 Gy (p=0.002) and 50 Gy (p=0.008) are statistically significant in favor of ${VMAT}_{I}$.

### B. Delivery differences

**Figure 5:**
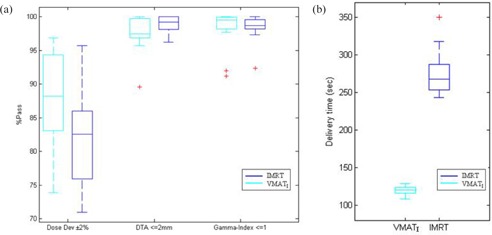
Box plot of: (a) Delta4 patient specific QA results (n=13), representing the median, maximum, minimum, and outliers for in‐house stringent criteria of DTA ≤2 mm and gamma index ≤1(±2%/±2 mm) with pass rate of 95% for ${\text{VMAT}}_{I}$ and IMRT plans; (b) delivery time (sec), ${\text{VMAT}}_{I}$ (single arc) vs. IMRT treatment plans for all 13 study cases.

In all 13 study cases, treatment beam delivery time (Fig. 5(b)) for ${\text{VMAT}}_{I}$ plans was reduced by more than 55% compared to IMRT plans (p=5.8E−10). IMRT plan delivery time ranged from 243 sec to 279 sec (median 254 sec). ${\text{VMAT}}_{I}$ delivery time ranged from 114 sec to 126 sec (median 120 sec). Monitor units delivered were reduced by up to 16% in ${\text{VMAT}}_{I}$ plans (median 313 MUs, range 271–381), compared to IMRT plans (median 369 MUs, range 309–448) (p=0.001).

### C. Dosimetry differences

All 13 cases tested through Delta4 QA phantom for dosimetric validation achieved 100% criteria of TG‐119^(6)^ gamma 3% and 3 mm. The in‐house stringent criteria, illustrated in the box plots (Fig. 5(a)), showed that ${\text{VMAT}}_{I}$ and IMRT plans satisfied the 95% pass rate criteria for gamma index ≤1 (max dose dev ±2% and max spatial dev ±2 mm) and DTA (≤2 mm). However, the dose deviation (±2%) showed greater variation in both ${\text{VMAT}}_{I}$ and IMRT plans. Dose deviation in IMRT, median 84.0% (range 71.1% to 95.7%) compared to VMAT plans, median 85.2% (range 76.9% to 89.7%).

### D. Single‐ vs. two‐arc VMAT plans

In this study, we also examined all 13 cases using single‐ vs. two‐arc VMAT (${VMAT}_{I}$ vs. ${VMAT}_{\text{II}}$). No significant difference in PTV coverage was observed: PTV D99 (p=0.76), PTV D95 (p=0.95), mean PTV dose (p=0.78), Van't Riet conformation number (CN) (p=0.26). Similarly, no statistically significant differences were found in OAR doses: heart V45 (p=0.78), heart mean dose (p=0.97), both lung V20 (p=0.77), lung V5 (p=0.93), and mean lung dose (p=0.86). However, we observed that the delivery time for two‐arc ${\text{VMAT}}_{\text{II}}$ plans increased almost two‐fold (${VMAT}_{I}$ delivery time median 120 sec, range 114 sec to 126 sec; ${\text{VMAT}}_{\text{II}}$ median 207 sec, range 207–251 sec; p=3.0E−11) compared to single‐arc ${VMAT}_{I}$. The median monitor units delivered in ${\text{VMAT}}_{I}$ was 309 MU, range 271–381 MUs, whereas for ${\text{VMAT}}_{\text{II}}$, the median was 332 MU and the range 298–487 MUs (p=0.1).

## IV. DISCUSSION

In this study, we selected the cases which had already been scanned and were treated with four‐or five‐field fixed‐beam step‐and‐shot IMRT. The dose distributions and dosimetric parameters of both IMRT and VMAT plans were clinically acceptable according to our clinical dosimetric criteria. Computation times for ${\text{VMAT}}_{I}$ and ${\text{VMAT}}_{\text{II}}$ optimization are generally longer than for IMRT. Longer computation times in VMAT treatment plan optimization is due to multiple steps in the overall process (e.g., initial arc creation, intensity modulation, arc sequencing, machine parameter optimization, adaptive dose convolution dose calculation, and segment weight optimization).^(9)^ Both ${\text{VMAT}}_{I}$ and ${\text{VMAT}}_{\text{II}}$ plans tended to be visually more homogenous and conformal with respect to PTV coverage compared with IMRT (Fig. 4 & Table 1). The differences in dosimetric parameters such as PTV mean dose, PTV D99, and PTV D95 are very small and not statistically significant. Similarly, for doses to organs at risk, there were no statistically significant differences found, except conformation (CN) number (p=0.04) and lung V20 (p=0.05), in favor of ${\text{VMAT}}_{I}$ plans as compared to IMRT plans. We also observed no statistical difference in dose distribution of ${\text{VMAT}}_{I}$ and ${\text{VMAT}}_{\text{II}}$ plans, except that the delivery time for ${\text{VMAT}}_{\text{II}}$ increased by two‐fold compared to ${\text{VMAT}}_{I}\;(p=3.0E-11)$.

Hawkins et al.^(10)^ also reported superior coverage of PTV and better sparing of the organs at risk with a VMAT technique. However, Hawkins and colleagues did their dosimetric comparison with forward conformal plans and a conformal arc (${\text{VMAT}}_{i}$). Our standard institutional protocol used in this study radically treats all patients having esophageal cancer using IMRT. Another difference from the Hawkins study was in the PTV generation. The PTV in our study was generated from an ITV created from motion information gained in the 4D CT. A uniform 0.5 cm expansion on the ITV is used to generate the PTV. The Hawkins study used a 1 cm standard equal margin around the CTV to create the PTV. The reported median lung V5 in our ${\text{VMAT}}_{I}$ plans was 60.4% (range 24%–68.2%), whereas Hawkins and colleagues reported a mean lung V5 of 51% (range 38%–84%) for ${\text{VMAT}}_{i}$ plans. Their study also reported the ${\text{VMAT}}_{i}$ maximum canal dose mean 29.4 Gy (range 21.7–38.7 Gy), whereas our observed ${\text{VMAT}}_{I}$ canal maximum dose was a median of 39.8 Gy (range 28.3–46.2 Gy).

A dosimetric study by Spencer et al.^(11)^ compared helical tomotherapy with IMRT and arc and two rapid arc with one arc. Spencer and colleagues reported improved plan quality and lower lung doses with two arcs compared to a single arc. We didn't identify any significant statistical difference in our study of two arcs compared with single arc for PTV coverage, nor for doses to organs at risk. However, we demonstrated that the cost of using VMAT two arcs is an increased delivery time and also an increased number of monitor units.

The findings in our study are also similar to some extent to those reported by Liam et al.^(12)^ Liam and colleagues did the IMRT planning retrospectively and their standard clinical plans were VMAT, whereas we began by using our standard clinical four‐ and five‐field IMRT plans and retrospectively generated VMAT plans to compare with the clinical IMRT plans. In our study, we were able to achieve better conformality and a visually better PTV homogeneity. The PTV dose coverage showed no significant hot or cold spots in the VMAT plans compared to the IMRT plans, whereas the Liam study describes a slightly better homogenous dose distribution for IMRT compared to VMAT. However, Liam and colleagues did not identify any conformality or homogeneity metrics that were used to demonstrate statistically significant differences between IMRT and VMAT planning techniques. The other major difference between these two studies is that in the Liam study, the PTV margins were dictated by the physician and varied case by case. In our study we used a 4D CT‐based ITV for the PTV generation.

We reported median statistics given concerns of the nonparametric nature of our data; therefore, direct comparison of dose metrics between these studies and ours is not possible.

In the current study, we examined the differences in integral dose, volume receiving different dose levels (2 Gy or more) between IMRT and VMATI plans, and phantom dosimetry validation for both IMRT and ${\text{VMAT}}_{I}$ techniques, which make our study unique among the recently published studies demonstrating the use of VMAT for esophageal cancers.

Concern of the increase in the integral dose in IMRT‐based treatment plans in the areas around the target (where the beams enter and exit) and also in areas far from the target still exist.^7^, ^8^, ^13^, ^14^, ^15^, ^16^ However, similar concerns regarding integral dose and a larger volume of normal tissue receiving low doses of radiation is also a concern in VMAT base planning. In this study, our results indicate that volume receiving low doses (≥2 Gy and up to 5 Gy) and the volume receiving 30 and 40 Gy were not improved in VMAT planning technique, as compared to IMRT. Whereas the volume receiving doses of 20 Gy (p=0.002) and 50 Gy (p=0.008) are statistically significant in favor of ${\text{VMAT}}_{I}$, although we used the same objectives for both IMRT and ${\text{VMAT}}_{I}$ plans. We concluded that the differences between the volumes receiving the different dose levels (2 Gy or more) in ${\text{VMAT}}_{I}$ might be due to the technique of ${\text{VMAT}}_{I}$ having a 360° arc (91 CPs). The dose conforms to a cylindrically shaped PTV volume, while minimizing the OAR doses (lungs, liver, and kidneys). As a result, it produced a distribution that is more like a parallel opposed pair distribution for the medium and lower dose levels (Fig. 4, transverse view) compared to the fixed field IMRT. Here the volumes receiving lower and medium dose levels are more likely due to exit and entry areas of the fixed beam angles.

Our use of 4D CT‐based ITV volumes also makes this study unique among the recent published studies demonstrating the use of VMAT for esophageal cancers. The importance of the 4D CT‐based ITV has already been demonstrated by several studies.^17^, ^18^, ^19^, ^20^ In this study, the helical voluntary exhale breath‐hold CT scan is used as primary CT dataset for volume delineation and dose computation. The CTV on 4D CT sequences and on primary helical CT scan were combined to form an ITV. This is reasonable, since the patient spends the majority of time in the exhale breathing phase. The challenge is that there is less imaging dose per phase in a 4DCT. This increases the imaging noise and makes it more difficult to contour the nodal volumes. Hence the helical scan is used for this. It has better soft‐tissue contrast and increased contrast‐to‐noise ratio, which makes contouring of the nodal targets easier, and it is the most conservative in terms of OAR DVH calculations. For example, the lung DVHs are computed with the minimum volume, making them a conservative overestimate.

## V. CONCLUSIONS

We demonstrate the feasibility and deliverability of VMAT plans in comparison with IMRT for the treatment planning and delivery for a large thoracic esophagus PTV volume surrounded by critical structures such as lungs, heart, liver, spinal canal, and kidneys. ${\text{VMAT}}_{I}$ combines the advantages of faster delivery and lower number of monitor units (MU), while maintaining the similar advantages of IMRT for conformal dose distributions to the target PTV and dose to the critical OARs. We also demonstrated that two‐arc ${\text{VMAT}}_{\text{II}}$ provides no significant benefit over single‐arc ${VMAT}_{I}$. Despite the benefits of VMAT‐based treatment techniques, we need to be cognizant of the potential implications of integral dose and a large tissue volume receiving low dose.

## ACKNOWLEDGMENTS

The authors would like to acknowledge the help of Erin Barnett for implementation of VMAT scripts in to the clinical treatment planning process as well as Dr. Ivan Yeung for the support of the project.
